# Development of a management protocol for internal carotid artery injury during endoscopic surgery: a modified Delphi method and single-center multidisciplinary working group

**DOI:** 10.1186/s40463-022-00582-w

**Published:** 2022-07-28

**Authors:** Amr F. Hamour, Frederick Laliberte, Vikram Padhye, Eric Monteiro, Ronit Agid, John M. Lee, Ian J. Witterick, Allan D. Vescan

**Affiliations:** 1 Department of Otolaryngology – Head and Neck Surgery, Temerty Faculty of Medicine, University of Toronto, Mount Sinai Hospital, 600 University Ave, Suite 401, Toronto, ON M5G 1X5 Canada; 2 Department of Otolaryngology – Head and Neck Surgery, Sinai Health System, Toronto, ON Canada; 3 Division of Neuroradiology, Joint Department of Medical Imaging, Toronto Western Hospital, University Health Network, Toronto, ON Canada; 4 Department of Otolaryngology – Head and Neck Surgery, St. Michael’s Hospital, Toronto, ON Canada

**Keywords:** Complications, Endoscopic surgery, Rhinology, Skull base, Endovascular treatment

## Abstract

**Background:**

Intra-operative internal carotid artery (ICA) injury during transnasal endoscopic surgery is a potentially catastrophic event. Such an injury is life-threatening in the immediate setting, with a reported peri-operative mortality rate of 10%. Nasal packing, muscle patches, direct vessel closure, and endovascular techniques have been described as useful strategies for managing ICA bleeds. The objective of this study was to develop a formalized management protocol for intra-operative ICA injury through engagement with a multi-disciplinary panel.

**Methods:**

A modified Delphi method including literature review, iterative rounds of stakeholder feedback, and expert panel discussions was used to develop a management protocol for ICA injury during transnasal endoscopic surgery. The 10-person multi-disciplinary panel included otolaryngologists, neurosurgeons, interventional neuroradiologists, anesthesiologists, and operating room nursing staff.

**Results:**

After three rounds of stakeholder engagement with the expert panel, consensus was reached on important elements to include within the protocol. The protocol was divided in three categories: Alert, Control, and Transfer. ‘Alert’ focusses on early communication with anesthesia and nursing staff. ‘Control’ focusses on techniques to expose the injury and obtain hemostasis or adequate tamponade. Lastly, ‘Transfer’ describes the process of contacting neuro-interventional radiology and safely transferring the patient. A one-page handout of the protocol was developed for placement in operating theatres.

**Conclusion:**

Due to the life-threatening nature of ICA injury, it is imperative that endoscopic sinus and skull base surgeons are prepared to manage this complication. Using a modified Delphi method with a multidisciplinary expert panel, a protocol for management of intra-operative ICA injury was developed.

**Graphical Abstract:**

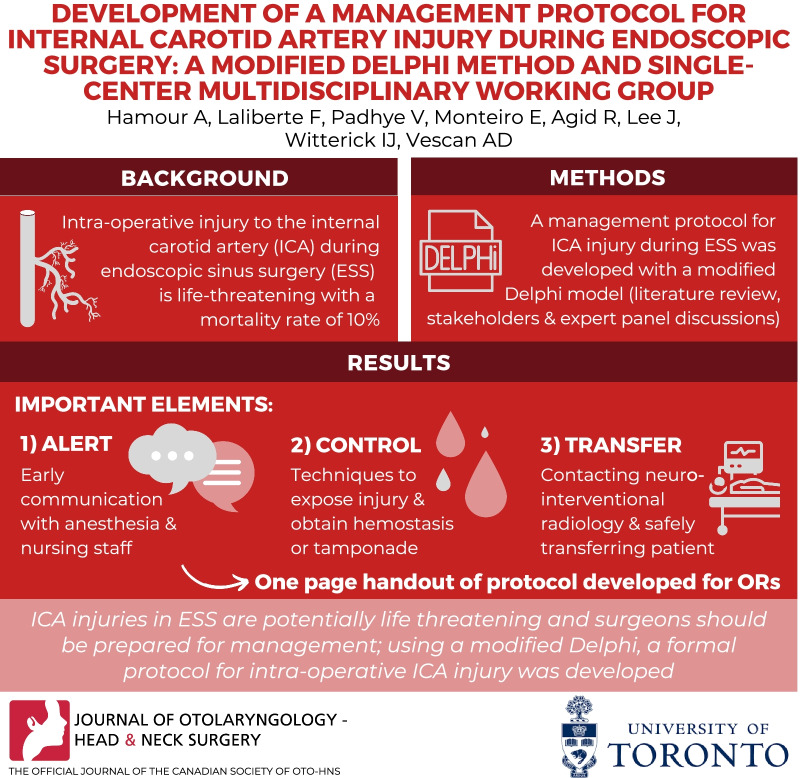

## Introduction

Global acceptance of transnasal endoscopic surgery for the management of sinus disease and skull base tumours has propelled this approach to the standard of care. Improved visualization, avoidance of skin incisions, and reduced duration of inpatient admission are a few of the many advantages that endoscopic approaches hold over traditional open approaches [1, 2]. Despite the positives, these approaches are not without risks. Both longer operative times as well as an increased incidence of cerebrospinal fluid (CSF) leaks have been attributed to endoscopic surgery [1]. In addition, intra-operative risk to important neurovascular structures, including the internal carotid artery (ICA), remain. Given the narrow anatomical view and need for specialized endoscopic instruments, intra-operative control of catastrophic bleeding can be uniquely challenging.

During routine endoscopic sinus surgery, incidence of ICA injury is exceedingly rare, often appearing in the literature as case reports [3]. In skull base cases utilizing an expanded endoscopic endonasal approach, the reported rate of ICA injury ranges from 0.4% to 3.4% [3, 4]. Such an injury is life-threatening in the immediate setting, with a reported peri-operative mortality rate of 10% [3, 5]. Of patients who survive past the immediate post-operative period, 88–90% live without permanent neurological deficits [3, 5]. This promising prognosis places emphasis on the surgical team to manage the intra-operative hemorrhage safely and effectively-in order to provide patients with the best chance at neurological recovery.

Currently, no formalized evidence-based management protocol exists for intra-operative ICA injury. Various factors including operative technique, choice of hemostatic agents, and team dynamics are important for obtaining adequate hemostasis [6]. Intra-operatively, nasal packing, muscle patches, direct vessel closure, and endovascular techniques have all been described as useful strategies for managing a catastrophic ICA bleed [6, 7]. Despite all of these options, there is no consensus on a superior strategy as each situation is unique. Therefore, it is important that the surgical team is prepared for even the most challenging situations. During times of stress, the importance of preparedness and teamwork become evident. Formalized protocols provide a way for teams to maintain organization and calmness during high intensity moments [8–10]. Often, they remind each team member of what tasks need to be completed within their role. When created using an evidence-based approach, formalized protocols can enhance team performance and improve patient outcomes [8, 11, 12].

Intra-operative ICA injury is a challenging, yet manageable complication of transnasal endoscopic surgery. Given the diversity of literature on management, a formalized protocol can be beneficial for surgical teams faced with this complication. The objective of this study is to develop a management protocol for intra-operative ICA injury using a modified Delphi method with engagement of a multi-disciplinary working group.

## Methods

### Study design

**Fig. 1:**
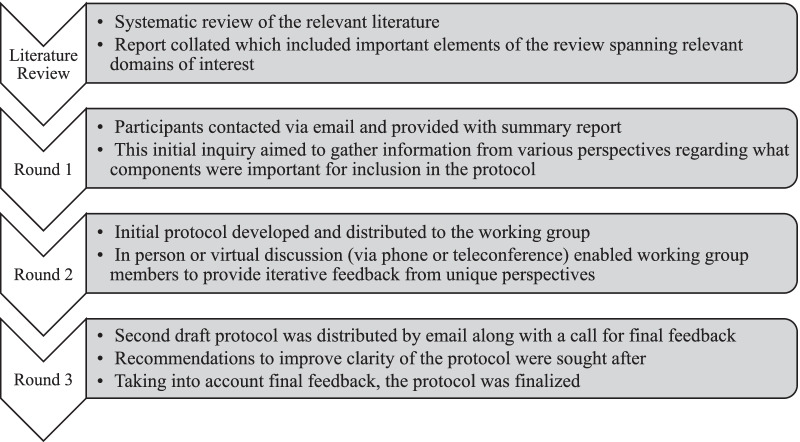
Modified Delphi method detailing study design

A modified Delphi consensus approach was used, guided by a systematic review of published work on management principles of intra-operative ICA injury during transnasal endoscopic surgery. Delphi methods are considered an appropriate means of obtaining consensus amongst a group of stakeholders. It was determined, a priori, that three rounds of stakeholder engagement would be undertaken (Fig. 1). Using two email based inquiries (rounds 1 and 3) and a one-on-one or small group virtual (telephone or teleconference) or in person discussion (round 2), members of the multi-disciplinary working group were able to provide feedback on the previous round in a controlled manner. For round 2, a nominal group technique was used to reach consensus. Following round 3, the protocol was finalized. Data were collected between February and June 2020.

### Literature review

A systematic review of literature relevant to endoscopic ICA injury management was undertaken using the following six databases (searches from inception to Feb 15 2020): MEDLINE, Scopus, PubMed, EMBASE, CINAHL, and the Cochrane database of systematic reviews. In addition, the following four clinical trial registries were searched: ClinicalTrials.gov, European Union Clinical Trials Register, International Clinical Trials Registry Platform, Cochrane Register of Controlled Trials. A list of relevant keywords related to the topic were determined by two authors (FL and AFH) and a combination of terms were utilized in the search strategy. Additional searching was performed including reviewing references of articles and citation searching. Eligibility of papers were assessed by FL and AFH, taking into account study design, outcome measures, and relevance to intra-operative management of endoscopic ICA injury. Case series describing events of ICA injury that did not focus on immediate intra-operative management were excluded. A summary of the review was provided as reference to members of the working group.

### Multi-disciplinary working group

Given the need for a dynamic team-based approach in managing intra-operative ICA injury, we sought to establish a multi-disciplinary working group composed of various important stakeholders. Potential working group members from the University of Toronto were identified by the authors (FL, AFH, and ADV) and invited via email to voluntarily participate. The final working group consisted of four endoscopic skull base surgeons (with representation from neurosurgery and otolaryngology—head & neck surgery), two anesthesiologists, two operating room nurses, and one interventional neuroradiologist.

### Round 1—Initial inquiry

Multi-disciplinary working group members were provided with the summary of the literature review via email. Their feedback was elicited via a set of closed and open-ended questions. The initial inquiry sought to establish the most important aspects of ICA injury management, from various perspectives. Given the diversity of experience of each working group member, questions addressing team dynamics, anesthesia management, and strategies for intra-operative control of hemorrhage were included.

### Round 2—In person/virtual discussion

An initial framework of the protocol was provided to each working group member prior to in person/virtual (phone or teleconference) feedback. This round enabled a free-flowing discussion enabling working group members to provide feedback on what they deemed most relevant to the protocol. Importantly, members were encouraged to provide recommendations specific to their area of expertise in addition to more general feedback.

### Round 3—Final feedback

The draft protocol was circulated to working group members in round 3. Final feedback was elicited via email with an emphasis on what variables to “keep” vs “remove.” Stylistic preferences and recommendations to improve the clarity of the protocol were encouraged. Again, working group members were encouraged to offer both broad and focused feedback specific to their clinical focus.

## Results

### Literature review

**Fig. 2:**
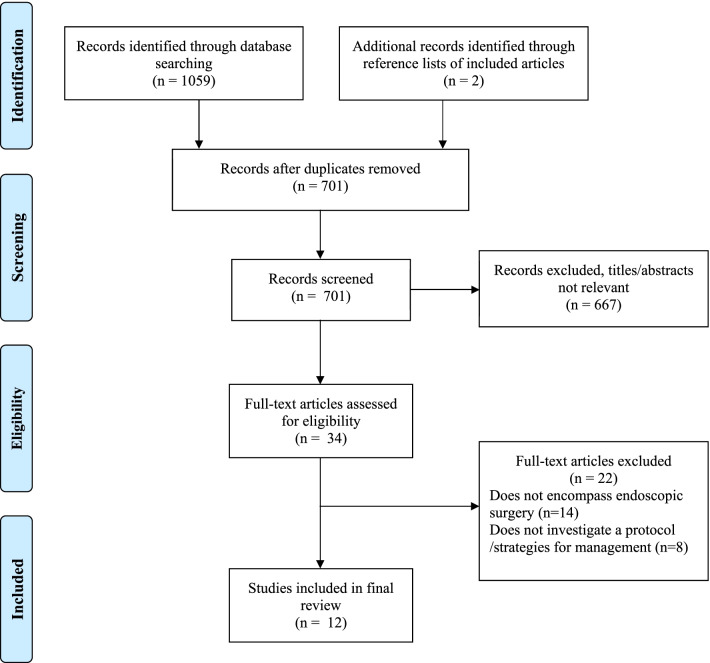
PRISMA diagram detailing study section process

The initial study searches yielded 1059 studies. Of these, 360 were duplicates. After reviewing the reference lists for articles undergoing review, 2 more studies were added resulting in 701 articles for screening (Fig. 2). Of these, 667 were excluded as the titles and/ or abstracts were not relevant to the study scope. This left 34 articles that met conditions for full-text review, which was carried out independently by two authors (AH and FL). In total, 22 articles were not relevant to the clinical question, leaving a final number of 12 articles ultimately included. Summary tables highlighting the studies were shared with the working group members.

### Delphi rounds

Initial email questionnaire to the working group yielded 7/9 responses. Representation from each specialty was observed. Members were asked to highlight the most important aspects of the protocol. A summary of included factors is presented in Table 1. Individuals were asked whether to “keep” or “remove” information about a particular topic/intervention in the protocol. Following this, a framework for the protocol was developed with input from the literature review and round one.

**Table 1:** Summary of potential protocol components presented to working group members

Category	Components
Communication	1. Call for assistance (from second surgeon, nursing staff, other healthcare providers)
	2. Clearly communicate to all members of the operating room team that ICA injury has occurred
	3. Seek advice from experienced skull base surgeon (if operator is inexperienced)
Surgical management	1. Recognize massive bleeding event
	2. Expose the injury to determine true source of bleed
	3. Use four hand technique where possible (with second surgeon)
	4. Use two large bore suction catheters (10 French or greater) to expose the injury while deflecting blood away from the camera
	5. Use crushed muscle patch (from anterolateral thigh or sternocleidomastoid) for packing
	6. Apply firm pressure for 10–12 min
	7. Request circulating nursing staff to bring all packing supplies into the operating theatre
	8. Consider direct endoscopic vessel closure if adequate intraoperative exposure
	9. Utilize hemostatic agents such as Gelfoam© (Pfizer, New York City), fibrin glue, oxidized cellulose packing, thrombin-gelatin matrix, and oxygel
	10. Utilize packing materials such as petroleum jelly-based gauze and foley catheter
Nursing considerations	1. Preoperative clarification of blood transfusion consent
	2. Have an understanding of where packing supplies are kept (preoperative)
	3. Have an understanding of where anesthetic supplies are kept (preoperative)
	4. Potential need for extra nursing support once injury has occurred
	5. Be prepared to aid anesthetist in resuscitation efforts
	6. Insert foley catheter
Anesthetic considerations	1. Preoperative anesthesia evaluation for high risk patients
	2. Perioperative preparedness of tests necessary prior to transfusion (i.e. group and screen)
	3. Call for help from second anesthetist to aid in resuscitation intraoperatively
	4. Obtain large bore intravenous access for resuscitation purposes
	5. Depending on circumstances, be prepared to initiate massive transfusion protocol
	6. Obtain arterial line to have real-time blood pressure measurements
	7. Administer tranexamic acid intravenously
	8. Understanding that the main goal is to maintain cerebral perfusion
Neuro-interventional radiology	1. Determination of local centers with capacity for endovascular interventions
	2. Alert neuro-interventional radiologist on call and describe need for urgent intervention
	3. Consideration of balloon occlusion test if patient is stable
	4. Consideration of a stent graft to seal the injury site and maintain cerebral blood flow
Considerations for transfer	1. Alert local transfer service (ambulance, helicopter, or other means)
	2. Prepare patient for transfer (leave intubated and secure lines)
	3. Primary surgeon should be available in case of secondary or residual bleed during transfer
	4. Take epistaxis tray and packing supplies during transfer
	5. Notify family members/ next of kin

In round 2, each working group member (9/9) provided feedback regarding the initial framework of the protocol. All members agreed to structure the protocol into three sections: (1) Alert (focused on communication and preparation), (2) Control (focused on surgical ICA injury management), and (3) Transfer (focused on transferring the patient to the appropriate tertiary care center). Members were provided the opportunity to provide feedback on what should be included and omitted in each relevant section. Input was collated and the protocol was structured as appropriate. Appropriate subheadings were included with relevant information in each section.

**Fig. 3:**
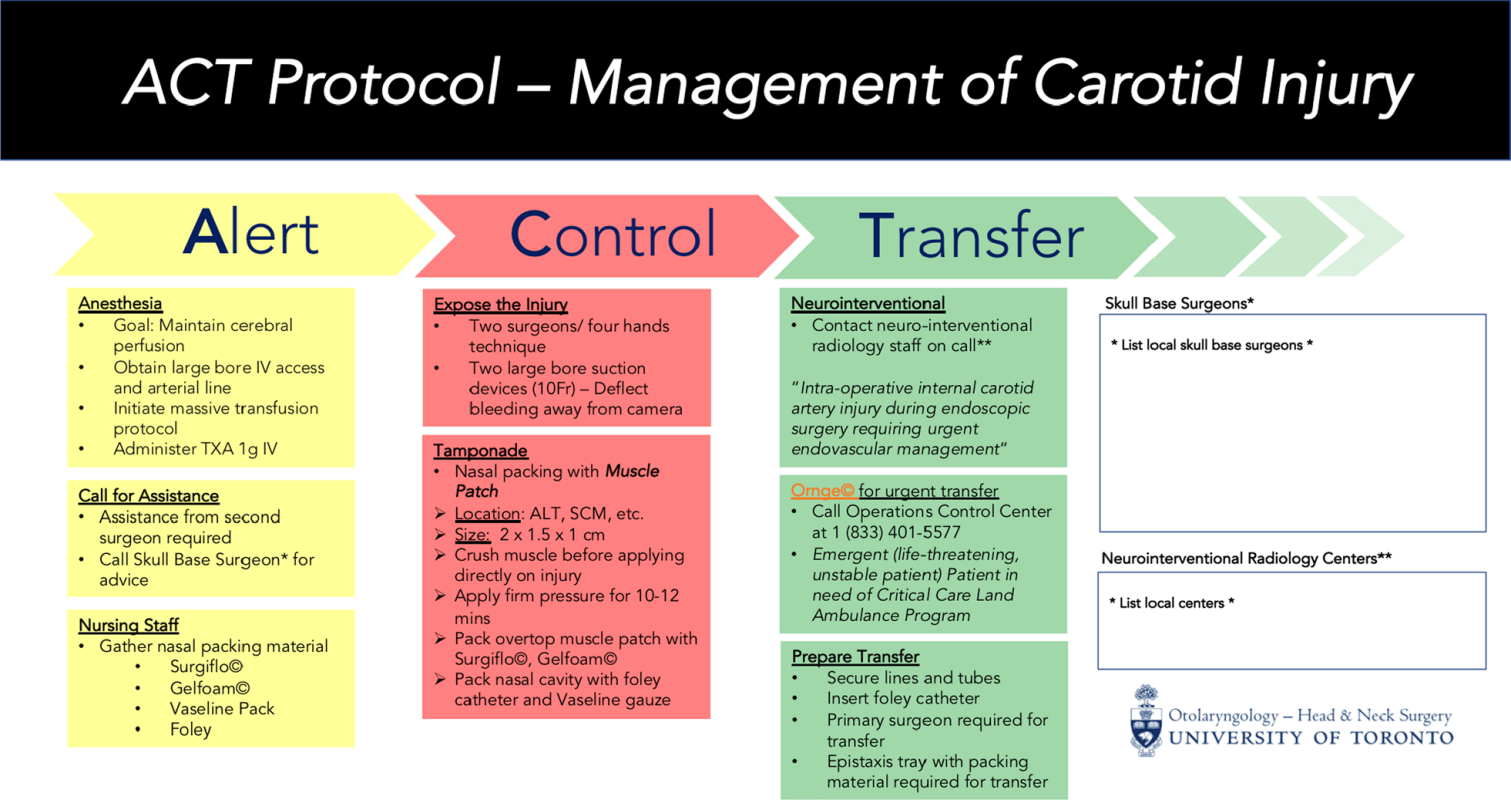
Final protocol for management of intra-operative internal carotid artery injury during endonasal endoscopic surgery. Orng© represents Ontario’s provincial medical transportation service for critically ill patients

The draft protocol was sent to each working group member in round 3. All members responded (9/9) and consensus was reached for all relevant variables in Table 1. Stylistic changes were made as appropriate to the final protocol (Fig. 3).

## Discussion

ICA injury during endoscopic sinus or skull base surgery is a devastating complication. Intra-operative and peri-operative management requires the surgical team to be well prepared. Given that ICA injury is a rare event, many surgeons have limited experience managing this complication should it occur [3, 4]. This study led to the development of a protocol (Fig. 3) to improve preparedness of surgical teams with variable levels of experience managing ICA injury.

A review of the literature revealed several important considerations that should be taken into account when managing ICA injury intra-operatively. Surgeons must promptly identify the injury and call for help from a surgical colleague as a four hand approach to managing this complication is optimal [6]. In addition, communication with colleagues in anesthesia is essential as major resuscitation may be necessary. Management of bleeding may be accomplished by use of a muscle patch, direct vessel closure, surgical clips, or bipolar electrocauterization, above other techniques [6, 7, 13]. Within the multi-disciplinary working group, the decision was made to organize the protocol in a chronological manner; guiding the team through the important considerations that must be considered. Regarding materials, emphasis was placed on the crushed muscle patch as the ideal option, in combination with various other packing adjuncts. Given the seriousness, ICA injury can induce panic and cause confusion within the operating theatre. A protocol could guide individual team members and promote a more coordinated effort in an otherwise potentially chaotic environment. Structured bleeding management protocols have been associated with improved patient outcomes [14]. The implementation of a bleeding management protocol in a cardiac surgery program was associated with significant reductions in the transfusion of allogeneic blood products, improved outcomes, and reduced cost [14]. Within the skull base literature, roughly 64% of centres have no formal ICA injury management protocol in place before first incidence of injury [3]. Standardized introduction of evidence-informed protocols at sites with high volume endoscopic endonasal surgery may provide teams with an improved capacity to manage this complication.

In high stress clinical scenarios, optimized team dynamics can be an important driver of success [12]. While a structured protocol may not change a clinical outcome, it provides a framework during a high-tension time period [8, 12]. The multi-disciplinary stakeholders included within this study were members who have direct involvement with rhinology and skull base patients. Hearing diverse perspectives from surgical, anesthesia, and nursing teams potentially reduces bias and provides a more holistic outlook. In addition, we discovered that navigating the hospital-to-hospital transfer process in order to obtain interventional neuroradiology services can be challenging in our local jurisdiction. Clarifying this becomes paramount when considering the high annual volume of endoscopic sinus surgery.

Managing challenging and time-sensitive complications in the operating theatre requires more than just a protocol for guidance. While a protocol may provide much needed support, it does not replace adequate training. Repeated exposure and practice in the form of surgical simulation can serve as an important education tool. In a recent survey of skull base surgeons who previously attended a course with vascular injury simulation, 20.6% of respondents reported an ICA injury in past 12 months [15]. While this relatively high rate may not represent the broader skull base community, it is insightful to appreciate that 50.9% of these injuries were associated with pituitary surgery with the parasellar carotid artery being the most commonly injured segment (39%) [15]. The same study found that when learning how to manage ICA injury, surgeons preferred live surgery, cadaver models, and animal models over computer-based simulation. Given that patient-related risk factors are the most common for ICA injury, practiced learning via the use of live models could serve as an important utility in training surgeons to manage these challenging situations [3]. Sheep models [16], three-dimensional printed synthetic models [17], and cadaver models have been described [18], with the latter having been associated with improved performance and reduced blood loss following repeat simulations.

There are several limitations to this study. Firstly, while the multi-disciplinary working group had a diverse range of expertise, we did not quantify the amount of experience each member has in managing this ICA injury intra-operatively. The systematic nature of our study protocol, focusing on literature review, was intended to tackle this potential lack of experience. Nonetheless, a larger expert panel with documented experience in managing ICA injury may have served to address this limitation. Further limitations included the size of our expert panel and that all members were from one institution. Given the relative rarity of ICA injury, establishing a larger working group from multiple centres would have increased the diversity of perspectives and may have ultimately improved the overall quality of the protocol. This point is underscored when reflecting on the expert-reliant nature of the study design. Lastly, it is important to highlight that the protocol developed in this study has not been validated. This study details the steps taken to design the protocol. Moving forward, incorporating this protocol in simulation-based workshops may help establish its external validity.

## Conclusion

Given the life-threatening nature of ICA injury, it is imperative that endoscopic sinus and skull base surgeons are prepared to manage this complication. This study utilized a modified Delphi method with a multidisciplinary working group to develop a protocol for management of intra-operative ICA injury.

## Data Availability

The datasets used and/or analysed during the current study are available from the corresponding author on reasonable request.
